# Histopathological and biochemical effects of green tea and/or licorice aqueous extracts on thyroid functions in male albino rats intoxicated with dimethylnitrosamine

**DOI:** 10.1186/1743-7075-6-2

**Published:** 2009-01-12

**Authors:** A Abd El Mgeed, M Bstawi, U Mohamed, M Abdel Gabbar

**Affiliations:** 1 Faculty of Science, Biochemistry Subdivision, Beni Sweif Branch, Cairo University, Cairo, Egypt; 2 Department of Physiology, Beni Sweif Branch, Cairo University, Cairo, Egypt

## Abstract

**Objective:**

To investigate histopathological and biochemical effects of green tea and/or licorice aqueous extracts in thyroid functions in male albino rats intoxicated with Dimethylnitrosamine.

**Methods:**

40 Male albino rats were divided into two main groups, 20 normal rats and 20 DMN intoxicated rats. Normal rats were subgrouped into 4 equal groups, group A without treatment (controls), group B treated with green tea, group C treated with licorice, group D treated with green tea and licorice. DMN intoxicated rats were subgrouped into 4 equal groups, group E treated with DMN, group F treated with DMN and green tea, group G treated with DMN and licorice, group H treated with DMN, green tea and licorice. The rats were permitted for free access to solubilized extracts of green tea and or licorice for 4 weeks. All rats in groups E, F, G, H were treated by intraperitoneal DMN (4 mg dissolved in 2.5 ml distilled water/kg body weight) seven times every 2 days in the first two weeks. Plasma total triiodothyronine and tetraiodothyronine were determined by radioimmunoassay. Thyroxine 5^-^monodeiodinase activity of liver was determined by spectrophotometeric method. Plasma thyroid stimulating hormone was determined by chemiluminometric technique. Histopathological examination was conducted.

**Results:**

Histopathologically thyroid gland of DMN intoxicated rats showed degeneration (DG)and desquamation (DS) of the lining epithelium and atrophy of many acini with hyperemia (H) in the stromal capillaries and In comparison with control, the administration of DMN alone induced decrease in plasma levels of T3 and T4 while it induced increase in plasma levels of TSH and hepatic activity of Thyroxine 5^-^monodeiodinase. Coadminstration of DMN and green tea attenuated the lowering effect of DMN on plasma levels of T3 and T4 and induced increase in these levels but values are still below normal ones while Co administration of DMN with licorice or mixture did not affect these levels. Co administration of green tea and/or licorice with DMN attenuated the rising effect of DMN on hepatic activity of Thyroxine 5^-^-DI while augmented the rising effect of DMN on plasma level of TSH.

**Conclusion:**

Aqueous extract of green tea may be effective in amelioration of biochemical effects and histopathological lesions induced by DMN.

## Introduction

Green tea the water extract of the dry leaves of the plant camellia sinensis, an evergreen shrub of the theaceae family, is a popular beverage commonly known as tea. A drink contains many compounds, including a mixture of polyphenols. Tea has been consumed by some human populations for many generations and, in some parts of the world, has been considered to have health – promoting potentials [1].

Glycyrrhiza glabra (Licorice) originated in the Mediterranean and Middle East has been used medically since at least 500 BC Ody [2]. It has been cultivated in Europe since at least the 16^th ^Century. It is sometimes known as the grand father of herbs [3].

Although a considerable body of information provides evidence supporting the preventive potential of green tea and licorice against diseases, proper understanding of the mechanisms by which tea and licorice reduce the risk of diseases is necessary.

As accumulative data were done by many previous authors [4,5] on the active ingredients of green tea and licorice. The recent work was performed to evaluate histopathological and biochemical effects of green tea and/or licorice aqueous extracts on thyroid functions in male albino rats intoxicated with Dimethylnitrosamine.

## Methods

Male albino rats weighing 150 g ± 20 were obtained from the animal house of Postgraduate and Research Institute, Alexandria University. The animals were kept in cages in environment with controlled temperature, humidity and illumination (24–25°C, 50–60% relative humidity,12 h light/dark cycle). Animals were put on basal diet and water supply ad libitum. 40 Male albino rats were divided into two main groups, 20 normal rats and 20 DMN intoxicated rats, normal rats were sub grouped into 4 equal groups, group A without treatment (controls), group B treated with green tea, group C treated with licorice, group D treated with green tea and licorice, DMN intoxicated rats were sub grouped into 4 equal groups, group E treated with DMN, group F treated with DMN and green tea, group G treated with DMN and licorice, group H treated with DMN, green tea and licorice. The animals were permitted for free access to solubilized extracts of green tea and or licorice for 4 weeks. All rats in groups E, F, G, H were given intra peritoneal DMN (4 mg dissolved in 2.5 ml distilled water/kg body weight) seven times every 2 days in the first two weeks. DMN doses injected to the animals were calculated according to Asakura et al., [6]. Chinese Green Tea was purchased from local market. Fresh tea was prepared by boiling 2.5 g of tea in 100 ml of boiling distilled water for extraction and the extract was administrated ad-libitum to animals of group B and group F (2.5% w/v) according to Bu – Abbas et al., [7] for 4 weeks. Licorice was purchased from local market. Fresh licorice was prepared by boiling 2.5 g in 100 ml of boiling distilled water for extraction and the extract was administrated ad-libitum to animals of group C and group G (2.5% w/v). Mixture of green tea and licorice was prepared by boiling 2.5 g of licorice and 2.5 g of tea in 100 ml of boiling distilled water for extraction and the extract was administrated ad – libitum to rats of group D and group H (2.5% w/v) for 4 weeks. After termination of the treatments the tested rats were sacrificed under diethyl ether anesthesia. Blood samples were collected from jugular vein in a little amount of 15% EDTA solution then centrifuged at 3000 r.p.m for 15 minutes where the clear non – haemolyzed supernatant plasma was quickly removed and kept at -20°C. Plasma levels of total T_3 _and T_4 _were determined by radioimmunoassay technique using Coat-A-count total T_3 _and T_4 _kits purchased from Diagnostic Products Corporation (DPC), Los Angeles. Plasma thyroid stimulating hormone level was determined by chemiluminometric technique using ACS:180 Automated Chemiluminescence's System. Liver tissues were quickly excised, weighed and homogenized in 0.9% saline solution and kept at – 20°C for the assay of thyroxine 5^- ^– monodeiodinase (5^- ^DI) [8] activity. For histopathological examination fixed specimens of thyroid gland were dehydrated in ascending ethanolic series, cleared in two changes of xylol and then impregnated and embedded in parablast at 60°C. Sections of 4–5 μm in the thickness, were prepared using a rotary microtone, hydrated using a descending series of alcohol after dewaxication, and stained later with hematoxylin and eosin. The stained sections were examined by a Zeiss(M35W) microscope as described by Drury [9].

The data of the present study are presented as Mean ± SD. The difference between groups was evaluated using ANOVA test and p < 0.05 was considered statistically significant.

## Results

Effect of green tea (G), Licorice(L) or their mixture on % of body weight increase, Liver weight/body weight and hepatic activity of thyroxine 5'-mondeiodenase (5'-DI), plasma levels of triiodothyronine (T_3_), tetraiodothyronine (T_4_), thyroid stimulating hormone (TSH) of normal and dimethylnitrosamine (D)-intoxicated male albino rats treated with green tea and/or licorice (L) are shown in Table (1) and Table (2), respectively.

**Table 1:** Effects of green tea (G), licorice (L) or their mixture on % body weight increase and % of liver weight/body weight of normal and dimethylnitrosamine (D)-intoxicated male albino rats.

Groups	Increase of B.W. %		Liver weight./body weight. %	
	
	Mean ± SE	% of change	Mean ± SE	% of change
Control	7.9 ± 0.228^c^		2.7 ± 0.267^c^	

D	15.3 ± 0.929^b^	+ 93.67	3.1 ± 0.218^abc^	+ 14.81

G	2.0 ± 0.045^e^	-74.48	2.9 ± 0.066^bc^	+ 7.40

L	1.7 ± 0.058^ef^	-78.48	2.8 ± 0.136^bc^	+ 3.70

G + L	2.6 ± 0.041^c^	-67.09	2.9 ± 0.196^bc^	+ 7.4

D + G	0.6 ± 0.17^f^	-96.08	3.1 ± 0.107^a^	0

D + L	27.8 ± 0.924^a^	+ 81.7	3.7 ± 0.314^ab^	19.35

D+G+L	-4.1 ± 0.171^d^	-126.80	3.4 ± 0.114^ab^	9.68

*Means that share the same superscript symbol are insignificantly different.

*% of changes of groups D, G and L are relative to control group.

*% of changes of groups D+G, D+L and D+G+L are relative to D group.

**Table 2:** Effect of green tea (G), licorice (L) or their mixture on levels of plasma thyroid stimulating hormone (TSH), triiodothyronine (T_3_), and tetraiodothyronine (T_4_), Thyroxine 5'-DI of normal and dimethylnitrosamine (D)-intoxicated male albino rats.

Groups	TSH (μIU/ml)		T_4 _(μg/dl)		T_3 _(ng/dl)		Thyroxine 5'-DI (ng hr^-1^/mg protein)	
	
	Mean ± SE	% of change	Mean ± SE	% of change	Mean ± SE	% of change	Mean ± SE	% of change
Control	0.09 ± 0.009^g^		31.7 ± 1.10^b^		792.7 ± 22.0^a^		1.47 ± 0.086^b^	

D	0.21 ± 0.003^f^	+133.33	7.1 ± 0.30^c^	-77.60	183.0 ± 5.70^d^	-76.91	1.89 ± 0.048^a^	+28.57

G	0.29 ± 0.010^c^	+ 222.22	34.1 ± 0.69^a^	+ 7.57	777.8 ± 26.4^a^	-1.88	1.21 ± 0.033^bc^	-17.69

L	0.20 ± 0.005^f^	+ 122.22	32.1 ± 0.74^b^	+ 1.26	610.4 ± 16.2^b^	-23.00	1.91 ± 0.105^a^	+29.93

G + L	1.35 ± 0.002^a^	+ 1400	29.3 ± 0.44^c^	-7.57	581.1 ± 18.1^b^	-27.69	0.96 ± 0.056^d^	-34.69

D + G	0.25 ± 0.007^d^	+ 19.05	12.3 ± 0.58^d^	+ 73.24	234.2 ± 8.7^c^	+27.98	1.19 ± 0.090^c^	-37.04

D + L	0.85 ± 0.016^b^	+ 305.76	7.7 ± 0.28^c^	+ 8.45	195.7 ± 7.5^cd^	+6.94	1.44 ± 0.113^b^	-23.81

D+G+L	0.23 ± 0.012^dc^	+ 9.52	8.2 ± 0.51^c^	15.49	195.6 ± 5.2^cd^	+6.89	0.78 ± 0.036^d^	-58.73

*Means that share the same superscript symbol are insignificantly different.

*% of changes of groups D, G and L are relative to control group.

*% of changes of groups D+G, D+L and D+G+L are relative to D group.

The general effect of % of body weight increase between groups was found to be very highly significant (p < 0.001). Green tea, licorice or mixture of both, produced a significant decrease in the % of body weight gain compared to normal control by 74.48%, 78.48% or 67.09%, respectively (Table 1). The increase in the % of the body weight gain produced by DMN was 93.67% compared to control group. Licorice administration to DMN-treated rats produced more increase in body weight gain by 81.70% while green tea or the mixture of those extracts produced marked decrease compared to DMN-administered rats by 96.08% or 126.80%. The general effect, in between groups, on % of liver weight/body weight was found by one way ANOVA to be significant (p < 0.05). Results indicated that green tea, licorice, DMN or the mixture exerted non significant change when compared to normal control group, (Table 1). The DMN groups administered with green tea, licorice or the mixture showed non significant changes compared to DMN control group, (Table 1). The general effect, in between groups, on the hepatic 5'-DI activity was found by one way ANOVA to be very highly significant (p < 0.001). Licorice or DMN exerted significant elevation in thyroxine 5'-DI level compared to normal control group by 29.93% or 28.57%, respectively (Table 2), while the mixture exerted significant decrease in that level by 34.69%. On the other hand, green tea caused non significant decrease in its level. Green tea, licorice or the mixture decreased significantly 5'-DI level of DMN-injected rats by 37.04%, 23.81% or 58.73%, respectively. The general effect, in between groups, on T_3 _level was found by one way ANOVA to be very highly significant (p < 0.001). Data showed that DMN, licorice or the mixture produced a significant decrease in plasma level of T_3 _compared to normal control group by 76.91%, 23.00% or 27.69%, respectively (Table 2), while green tea exerted non significant change in its level. Licorice or the mixture exerted no significant changes in the depleted T_3 _level of DMN-injected rats, although green tea produced a significant elevation in its level by 27.98%. The general effect, in between groups, on T_4 _level was found by one way ANOVA to be highly significant (p < 0.01) while green tea produced a significant elevation in its level compared to normal control group by 7.57% (Table 2). DMN or the mixture exerted a significant decrease in its level by 77.60% or 7.57% respectively. On the other hand, licorice exerted non significant change in its level. The treatment of DMN-injected animals with green tea induced a significant increase in that level by 73.24%, while licorice or the mixture exerted non significant changes in its level. The general effect, in between groups, on TSH level was found by one way ANOVA to be very highly significant (P < 0.001). Green tea, licorice, DMN or the mixture caused a significant elevation in TSH level compared to normal control group by 222.22%, 122.22%, 133.33% or 1400.0%, respectively (Table 2). Green tea, licorice or combination of both produced more increase of its elevated level of DMN-injected rats by 19.05%, 305.76% or 9.52%, respectively.

**Figure 1:**
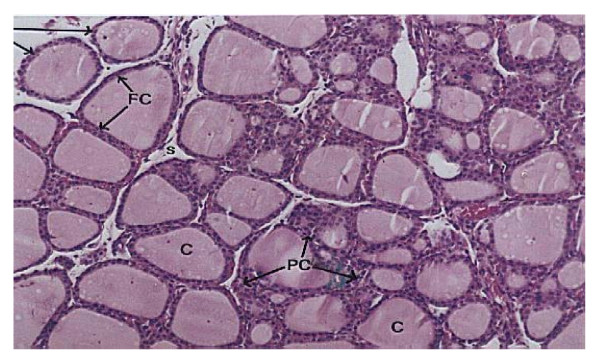
**Normal thyroid tissue of a control rat(H × &E stain ×128)**.

**Figure 2:**
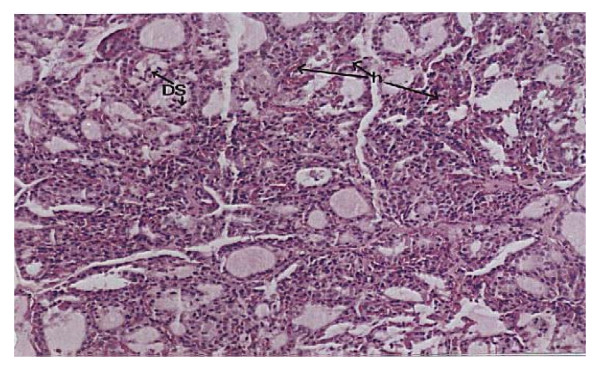
**Thyroid tissue of a rat intoxicated with DMN (H × & E stain × 125)**.

**Figure 3:**
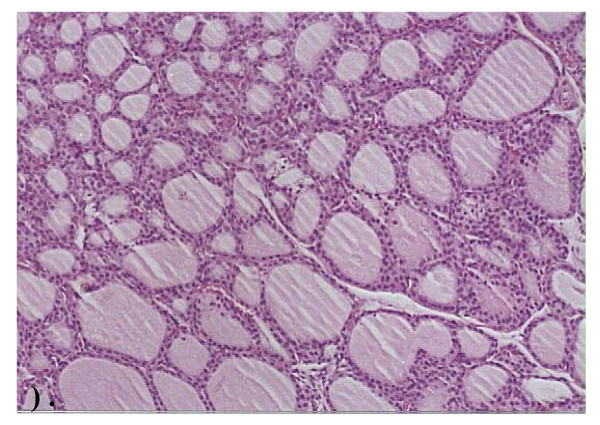
**Thyroid tissue of a rat intoxicated with DMN treated with a mixture of green tea and licorice (H × & E stain ×125)**.

**Figure 4:**
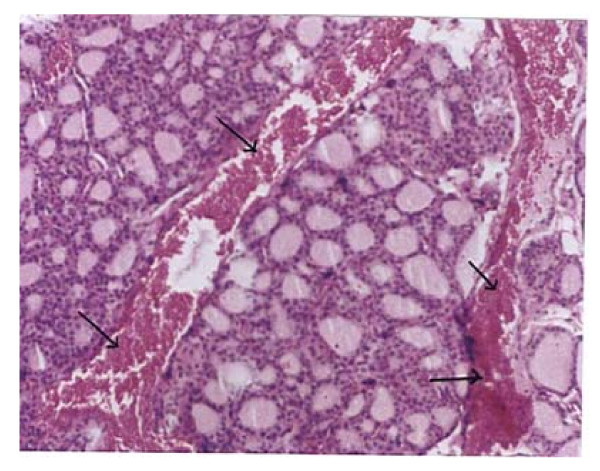
**thyroid tissue of a rat intoxicated with DMN for 2 weeks and treated with green tea for 4 weeks (H × & E stain ×128)**.

**Figure 5:**
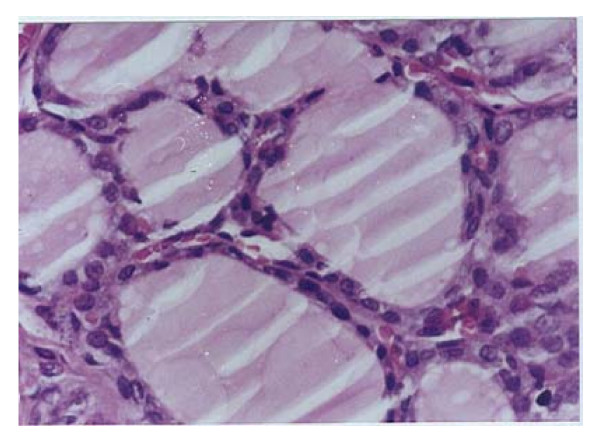
**Thyroid tissue of a rat intoxicated with DMN and treated with licorice (H × & E stain × 512)**.

**Histopathological examination**, Figure (1) demonstrates the normal thyroid tissue of a control rat. It is organized into follicles (F) filled with moderate amount of colloid (C)in their lumen and lined with cuboidal epithelial follicular cells (FC). The follicular acini are separated by follicular spaces or septa (S). Some parafollicular cells(PC) are present in between thyroid follicles. Figure (2) demonstrates the thyroid tissue of a rat intoxicated with DMN showing degeneration and desquamation (DS) of the lining epithelium and atrophy of many acini associated with hyperemia (h) in the stromal capillaries. Figure (3) demonstrates the thyroid tissue of a rat intoxicated with DMN and treated with a mixture of green tea and licorice showed marked amelioration of the lesions in thyroid tissue with enlargement of thyroid follicles. Figure (4) demonstrates the thyroid tissue of a rat intoxicated with DMN for 2 weeks and treated with green tea for 4 weeks showing focal hemorrhage (arrow) in the intracinar blood vessels. Figure (5) demonstrates The thyroid tissue of a rat intoxicated with DMN and treated with licorice showing great amelioration and normalization of thyroid tissue architecture.

## Discussion

Body weight is frequently the most sensitive indicator of the adverse effects of xenobiotics, so it is considered as a determined parameter of toxicity testing. Administration of tested extracts decreased body weight gain in normal animals, in agreement with other researchers [10,11] who reported that green tea suppressed the body weight gain as well as the gain of intraperitoneal adipose tissues. However, DMN enhanced that gain. Administration of licorice to DMN-injected animals augmented DMN effect by enhancing that gain, while the mixture or green tea markedly attenuated that effect. This change may be related to the hypertriglyceridemic effect of green tea, which caused the release of lipids from their depots, liver and adipose tissues, and its lowering effect on weight of various adipose tissue [11,12]. Administration of tested extracts and DMN had insignificant increase in liver weight/body weight % ratio. The increase was more pronounced in DMN-injected rats. Administration of green tea showed no effect on marked increase induced by DMN intoxication on that ratio, while licorice or mixture augmented this effect. So, it can be concluded that DMN might cause mild liver hypertrophy. However, while licorice or the mixture enhanced that effect, green tea exerted no change on it. In view of the present study, DMN caused substantial decreases in plasma levels of T_3 _and T_4 _concentrations consistent with a tremendous increase in plasma level of thyroid-stimulating hormone (TSH). DMN also increased the hepatic activity of thyroxine 5'-monodeiodinase (thyroxine 5'-DI). Thus, the stimulatory effect of DMN on 5'-DI was not accompanied with an increase of plasma T_3 _level. Not only the hepatic 5'-DI activity, but also the continuous release from thyroid gland and uptake of the hormone by the tissues can control T_3 _level in plasma [13]. Based on this assumption, it is worth mentioning that the decreased level of plasma T_3 _concentration in DMN-injected animals may be attributed to a decreased secretion and/or an increased uptake or extraction of the hormone by the tissues. Moreover, in parallel with the decrease in T_3 _and T_4 _levels in DMN intoxicated animals pathological examination of thyroid tissue, showed degeneration, desquamation and atrophy of many acini as indicated in recent study. Green tea decreased T_3 _level, although that decrease was statistically insignificant, while it increased the plasma levels of TSH and T_4_. On the other hand, green tea attenuated the lowering effect of DMN on T_3 _and T_4 _levels by increasing these levels, the values are still below the normal control ones. In parallel with amelioration of T_3 _and T_4 _levels induced by green tea administration in respect to DMN lowering effect histopathological examination revealed that green tea improved the thyroid perturbations produced by DMN intoxication. Green tea profound the DMN rising effect on TSH level by inducing a slight increase in its level. This is logically accepted because green tea antagonized DMN effect and increased T_3 _and T_4 _levels. Green tea induced a decrease in hepatic thyroxine-5'-DI activity but it was statistically insignificant. Also, green tea suppressed the rising effect of DMN on 5'-DI activity by decreasing it, so it is suggested that green tea decreased the rate of conversion of T_4 _into T_3 _and the increase of T_3 _and T_4 _levels may be attributed to increased secretion and the improvement of thyroid tissue architecture. These observations agree with findings reported by Satoh *et al*[14]., Licorice induced a decrease in T_3 _level, while it had no significant change on T_4 _level. On the other hand, licorice induced a substantial increase in TSH level. Licorice profound the elevated effect of DMN on TSH level. It was remarkable that the effect of licorice and DMN on TSH level was additive. The co-administration of DMN and licorice did not affect the lowering effect of DMN on T_3 _and T_4 _levels. Licorice induced an increase in thyroxine 5'-DI activity, but it attenuated the rising effect of DMN on that activity. Linking rise of 5'-DI activity induced by licorice with its lowering effect on T_3 _level, showing an impression that the stimulated hepatic 5'-DI may be stressed to acclimatize the decrease of T_3 _level, a kind of compensatory mechanism. Consistent with these results, Martinez-deMena *et al.*,[15] stated that T_3 _up regulates 5' DI (type II) in brown adipose tissues. It was obvious that the tremendous increase in TSH level exerted by the mixture was a result of synergistic effect between licorice and green tea. The increase in TSH level was concomitant with the decrease of T_3 _and T_4 _levels by the mixture. Surprisingly, although the mixture profound the DMN rising effect on TSH level, the increase was not additive. On the other hand, the co-administration of the mixture with DMN did not significantly affect the lowering effect of DMN on T_3 _and T_4 _levels. The mixture decreased thyroxine 5'-DI activity and inhibited the rising effect of DMN on that activity.

In conclusion, these results indicated that administration of green tea alone in DMN-injected animals was more pronounced in increase of T_3 _and T_4 _levels than concomitant administration of green tea and licorice. Also this study revealed that the treatment of the DMN intoxicated animals with green tea and/or licorice induced marked amelioration of pathological lesions induced in thyroid tissue manifested by increase in the number and size of follicles which become more organized and appeared to have higher amount of colloid.
